# Frequency of Abnormalities Detected by Point-of-Care Lung Ultrasound in Symptomatic COVID-19 Patients: Systematic Review and Meta-Analysis

**DOI:** 10.4269/ajtmh.20-0371

**Published:** 2020-06-02

**Authors:** Mouhand F. H. Mohamed, Shaikha Al-Shokri, Zohaib Yousaf, Mohammed Danjuma, Jessiya Parambil, Samreen Mohamed, Mahmood Mubasher, Mujahed M. Dauleh, Bara Hasanain, Mohamed Awni AlKahlout, Ibrahim Y. Abubeker

**Affiliations:** 1 Internal Medicine Department, Hamad General Hospital, Hamad Medical Corporation, Doha, Qatar;; 2 Unity Hospital of Rochester, Rochester, New York;; 3 Nephrology Department, PennState Hershey Medical Center, Hershey, Pennsylvania;; 4 Saint Agnes Hospital, Baltimore, Maryland

## Abstract

The COVID-19 pandemic has resulted in significant morbidity, mortality, and strained healthcare systems worldwide. Thus, a search for modalities that can expedite and improve the diagnosis and management of this entity is underway. Recent data suggested the utility of lung ultrasound (LUS) in the diagnosis of COVID-19 by detecting an interstitial pattern (B-pattern). Hence, we aimed to pool the proportion of various reported lung abnormalities detected by LUS in symptomatic COVID-19 patients. We conducted a systematic review (PubMed, MEDLINE, and EMBASE until April 25, 2020) and a proportion meta-analysis. We included seven studies examining the role of LUS in 122 COVID-19 patients. The pooled proportion (PP) of B-pattern detected by lung ultrasound (US) was 0.97 (95% CI: 0.94–1.00 *I*^2^ 0%, *Q* 4.6). The PP of finding pleural line abnormalities was 0.70 (95% CI: 0.13–1.00 *I*^2^ 96%, *Q* 103.9), of pleural thickening was 0.54 (95% 0.11–0.95 *I*^2^ 93%, *Q* 61.1), of subpleural or pulmonary consolidation was 0.39 (95% CI: 0.21–0.58 *I*^2^ 72%, *Q* 17.8), and of pleural effusion was 0.14 (95% CI: 0.00–0.37 *I*^2^ 93%, *Q* 27.3). Our meta-analysis revealed that almost all SARS-CoV-2–infected patients have abnormal lung US. The most common abnormality is interstitial involvement depicted as B-pattern. The finding from our review highlights the potential role of this modality in the triage, diagnosis, and follow-up of COVID-19 patients. A sizable diagnostic accuracy study comparing LUS, computed tomography scan, and COVID-19–specific tests is warranted to further test this finding and to delineate the diagnostic and prognostic yield of each of these modalities.

## INTRODUCTION

The COVID-19 pandemic has put enormous pressure on healthcare systems all around the globe.^1^ Since its advent, there has been a quest for aiding symptoms, signs, laboratory, and imaging modalities that assist in triaging and prioritizing patients for testing and isolation. This is of exceptional value when dealing with atypical presentations of COVID-19 or when working in resource-depleted settings. Computed tomography (CT) scan of the chest has emerged as the imaging modality of choice in the diagnosis of this disease.^2^ The main findings are that of interstitial involvement.^2^ However, the difficulties associated with the transfer of infectious and potentially sick patients, disinfecting the machine, ionizing radiation exposure, immediate availability concerns, and the need for lesions follow-up made it less appealing as a triaging tool for clinicians, especially those working in the front line.^3,4^

Lung ultrasound (LUS) or point-of-care ultrasound (POCUS) has gained popularity in the triage, diagnosis, and follow-up of various lung lesions and is considered an alternative to chest X-ray (CXR) and CT scan.^5,6^ It is used routinely by critical care specialists, emergency physicians, and, recently, internists.^7^ It demonstrated a better diagnostic yield than a CXR in the early diagnosis of H1N1 2009 pandemic viral pneumonia.^8^ Recent data suggested the potential utility of lung US in the diagnosis of COVID-19, depicting interstitial phenomenon as evident by B-lines.^4,9,10^ Lung US is a tempting modality, given the ease of use, availability in many emergency departments, relative ease of disinfection, and potential role in the follow-up.^11,12^ Thus, we aimed to explore the potential utility of this modality by systematically reviewing the literature and describing the frequency of B-pattern detected by lung US. In addition, we describe the frequency of other lung abnormalities detected by this modality.

## METHODS

This is a systematic review and a meta-analysis keeping with PRISMA guidance.^13^

### Study eligibility criteria.

We included case series and observational studies guided by the following inclusion criteria:

1. Adult patients older than 18 years.
2. Confirmed COVID-19 infection.
3. The index test is LUS.
4. The study reports the frequency of abnormalities detected by LUS.

### Exclusion criteria.

1. Studies performed solely on asymptomatic patients.
2. No clear description of LUS abnormalities or their frequencies.
3. Studies evaluating pediatric population.
4. Studies not meeting the inclusion criteria.

### Search strategy.

We performed a comprehensive literature search of PubMed, Medline, and EMBASE since their inception, with no limitations. The search was updated on April 22, 2020. Example of a database search strategy is as follows: (“LUS” OR “point of care ultrasound” OR POCUS OR ultrasound OR “ultrasound”/exp/mj OR “point of care ultrasound”/exp/mj) AND (“COVID-19” OR (sars AND cov AND 2) OR “COVID 19” OR “covid 19”/exp/mj OR “COVID-19”/exp/mj). Besides, we performed a manual reference search and free-text search on Google and Google Scholar to further add to the search comprehensiveness.

### Screening and data extraction.

Initial title and abstract screening were conducted by two reviewers (M. F. H. M. and S. A.). Potentially eligible articles were imported for full-text review and assessed for inclusion. A third reviewer (I. Y. A.) adjudicated discrepancies guided by the protocol whenever disagreement arose that was not settled by discussion. We extracted data using an Excel sheet. Examples of data collected are author, year of publication, study type, type of probe, frequency of various lung abnormalities, and the severity of the illness.

### Outcome.

We performed a scoping search and reviewed some of the constituent studies to identify the commonly reported outcomes.^10^ This was done at the design phase before proceeding with the actual search. We opted to summarize the pooled proportion (PP) of various lung abnormalities detected by LUS. These abnormalities are as follows:

1. B-pattern (positive if three or more B-lines were present in a lung region, confluent B-lines, or white lung appearance).
2. Pleural line abnormalities: Some of the constituent studies did not use a uniform description when referring to pleural changes. Hence, we pooled the higher frequency of either pleural thickening or pleural line irregularities.
3. Pleural thickening was solely pooled.
4. Consolidations: The reporting of consolidation was incomplete. So, we chose to combine subpleural and pulmonary consolidations and considered the higher frequency of the two.
5. Pleural effusion.

### Study quality and risk of bias assessment.

We used the QUDAS 2 quality assessment score to judge the quality of the included studies in our review.^14^

### Statistical analysis.

We used a proportion meta-analysis to summarize or pool the frequency of various findings on lung US (based on our scoping review, we concluded that the sensitivity, specificity, and diagnostic accuracy could not be computed from the constituent studies). We used the random-effects model (double arcsine transformation and back transformation). *I*^2^ was used to adjudicate heterogeneity (> 50% was considered marked). The analysis was conducted via MetaXL version 5.3 (EpiGear International, Sunrise Beach, Queensland, Australia).

## RESULTS

**Figure 1.**
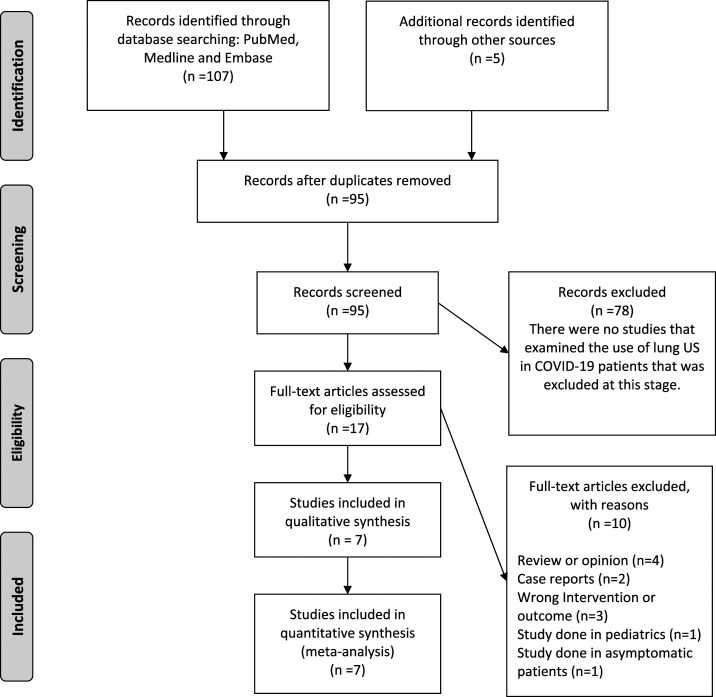
Flow diagram.

Our initial database search has retrieved 107 potentially relevant articles. Finally, after duplicate removal and full-text screening (all articles excluded were duplicates, reviews, opinions, or case reports), seven articles were included in our quantitative synthesis (Figure 1 flow diagram).^9,10,12,15–18^ A total of six observational studies and a case series describing a total of 122 patients constituted our review population (Table 1 presents a summary of the included studies).

**Table 1:** Summary of studies included in the systematic review

Author	Study design	Number of patients	Disease severity/setting	Probe used/number of zones scanned	B-pattern frequency	Bilateral B-pattern (interstitial syndrome)	Consolidation frequency	Thickened pleura line	Pleural line abnormalities (thickening or irregularities)	Pleural effusion frequency	Subpleural consolidations
Lomoro et al. 2020	Retrospective	22 (males % NS)	NS/ED	Linear or convex/NS	B-pattern 100% (*n* = 22/22)	Bilateral various B- patterns in 100% (*n* = 22/22)	NS	13.6% (*n* = 3/22)	Thickened 13.6% (*n* = 3/22)	4.5% (*n* = 1/22)	27.3% (*n* = 6/22)
Peng et al. 2020	Retrospective	20	NS/NS	NS/12 zones	B-pattern 100% (*n* = 22/22)	Bilateral B-pattern 75% (*n* = 15/20)	NS	Most patients (NS)	NS	NS	NS
Yi 2020	Retrospective	20 (males 55%)	NS/inpatient (NS)	Linear or convex/12 (BLUE protocol)	B-pattern 100% (*n* = 20/20)	NS	75% (*n* = 15/20)	Pleural thickening 35% (*n* = 7/20)	Unsmooth or interrupted pleural line 100% (20/20)	60% (*n* = 12/20)	NS
Poggiali et al. 2020	Retrospective	12 (males 75%)	None severe/ED	NS/NS	Diffuse B-pattern with spared areas 100% (*n* = 20/20)	NS	–	NS	NS	NS	Posterior 25% (*n* = 3/12)
Lu et al. 2020	Retrospective	30 (males 53%)	50% severe or critical/inpatients (NS)	Linear or convex/12 zones	B-pattern 90% (*n* = 27/30)	Bilateral 73% (22/30)	Pulmonary consolidation 20% (*n* = 6/30)	Pleural thickening (no serrated margins) 10% (*n* = 3/30)	Pleural thickening (no serrated margins) 10% (*n* = 3/30)	3.3% (*n* = 1/30)	NS
Lyu 2020	Retrospective	8 (males 25%)	Severe or critical/inpatients, otherwise, NS	Convex array probe/10 zones (modified BLUE protocol)	B-pattern (white lung appearance) 100% (*n* = 8/8)	NS	Pulmonary or suppleural 37.5% (*n* = 3/8)	Pleural thickening 100% (*n* = 8/8)	Pleural thickening 100% (*n* = 8/8) and blurry or irregular fragmentation 75% (6/8)	12.5% (*n* = 1/8)	Pulmonary or subpleural 37.5% (*n* = 3/8)
Yasukawa and Minami 2020	Retrospective	10 (males 70%)	Mod-severity/inpatient	Phased array probe/12 zones	Glass rockets (> 5 B-lines) 100% (*n* = 10/10) and confluent B-lines 100% (*n* = 10/10)	NS	Consolidation 10% (1/10)	Thick irregular pleural line 100% (*n* = 10/10)	Thick irregular pleural line 100% (*n* = 10/10)	0% (*n* = 0/10)	50% (*n* = 5/10)

ED = emergency department; ICU = intensive care unit; NS = not specified in the study.

### Lung zones examined.

Five studies reported on the number of lung zones examined. Twelve zones were examined in four studies, whereas in one study, 10 zones were examined (Table 1).

### The proportion of B-pattern.

**Figure 2.**
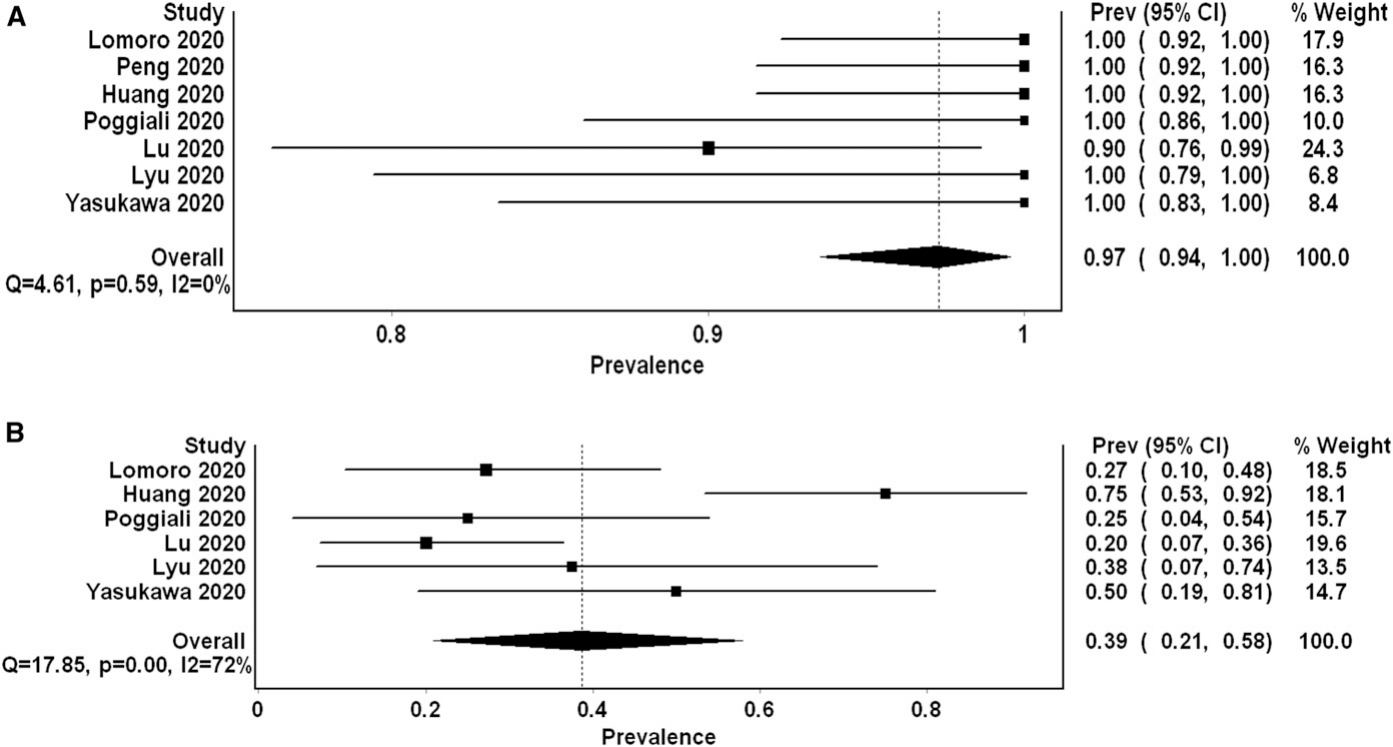
Forest plot presenting (**A**) the pooled proportion of B-pattern and (**B**) consolidation (the higher frequency of subpleural or pulmonary consolidations reported by the primary study) detected by lung ultrasound in symptomatic COVID-19 patients. *I^2^ is 0% for B-pattern proportion, suggesting homogeneity of data. There is marked heterogeneity depicted by extremely high *I*^2^ for the finding of consolidation.

All seven studies reported on the frequency of B-pattern. The PP of B-pattern in the review population is 0.97 (95% CI: 0.94–1.00 *I*^2^ 0%, *Q* 4.6). The results were homogenous and consistent among studies (Figure 2).

### The proportion of pleural line abnormalities.

**Figure 3.**
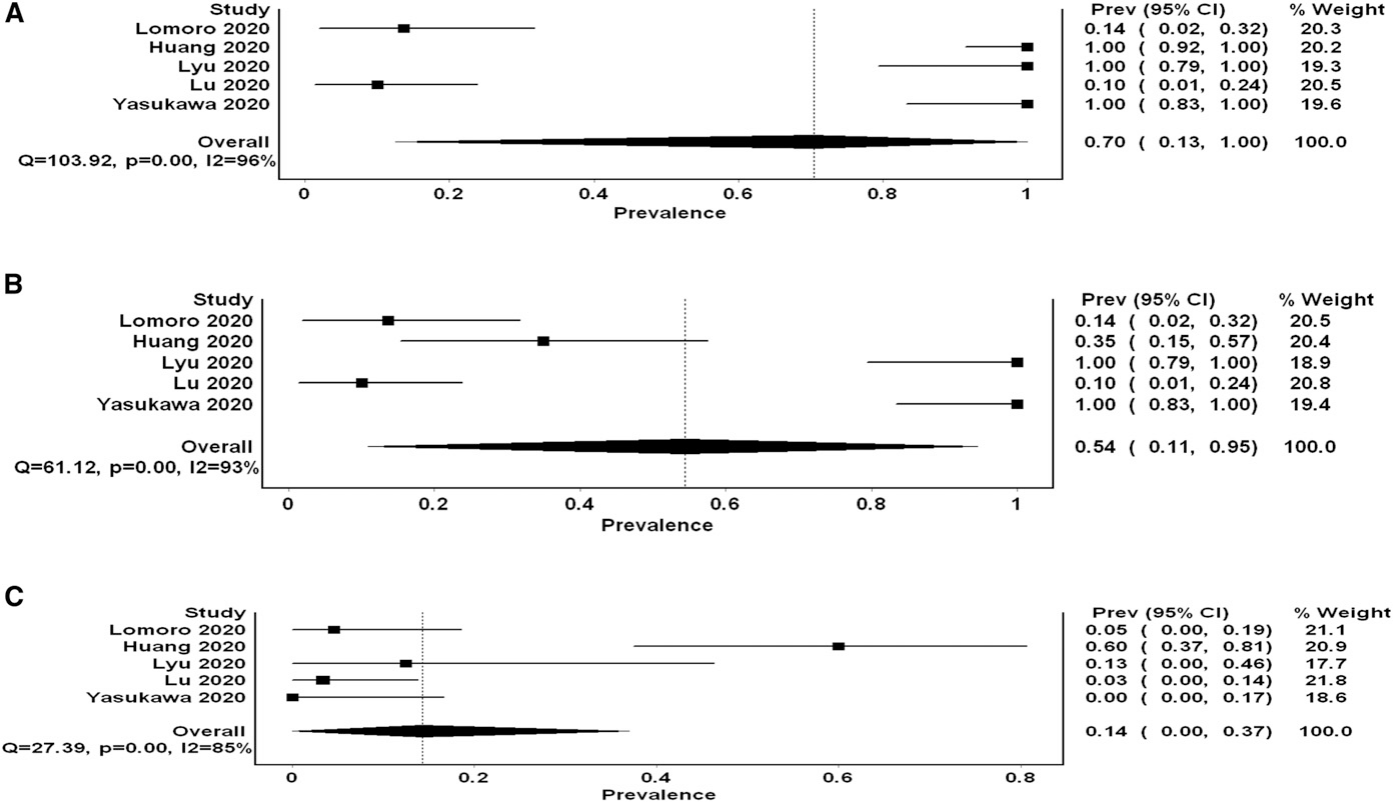
Forest plot depicting (**A**) the pooled proportion of pleural line abnormalities (pleural thickening or irregularities, whichever is higher), (**B**) pleural thickening, and (**C**) pleural effusion detected by lung ultrasound in symptomatic COVID-19 patients. * There is a high heterogeneity depicted by extremely high *I*^2^ for all three abnormalities.

Five studies reported the frequency of pleural line abnormalities. The frequency of these abnormalities ranged between 10% and 100% (Table 1). The PP is 0.70 (95% CI: 0.13–1.00 *I*^2^ 96%, *Q* 103.9) (Figure 3). One study did not report the exact frequency but stated that most patients had pleural thickening; hence, pooling this additional study may have led to a slight increase in the PP.

### The proportion of pleural thickening.

Five studies reported the frequency of pleural thickening. The PP of pleural thickening is 0.54 (95% 0.11–0.95 *I*^2^ 93%, *Q* 61.1). This was less than the PP of pleural line abnormalities because of one study that reported a 35% frequency of pleural thickening while reporting a 100% frequency of pleural line irregularities. There was marked heterogeneity evident by the high *I*^2^ (Figure 2). As explained earlier, the study by Peng et al.^10^ reported that pleural thickening has occurred in most patients without specifying the frequency, hence excluded from the computation of this PP.

### The proportion of consolidations.

Six studies reported on the frequency of subpleural or pulmonary consolidations detected by lung US. The frequency ranged from 20% to 75%. The PP is 0.39 (95% CI: 0.21–0.58 *I*^2^ 72%, *Q* 17.8) (Figure 2). *I*^2^ indicated a marked heterogeneity.

### The proportion of pleural effusion.

Five studies reported the frequency of pleural effusion detected by lung US. It ranged from 0% to 12.5% in four studies, whereas in one study, 60% of the patients had pleural effusion. The PP of this finding was the lowest at 0.14 (95% CI: 0.00–0.37 *I*^2^ 93%, *Q* 27.3). The results of these studies were significantly heterogeneous (Figure 3).

### Risk of bias assessment.

The funnel plot depicted moderate to marked asymmetry, suggesting potential publication bias (Supplemental Figure 1). For comparison, we populated DOI plots with LFK indices to ascertain the publication bias. Using these additional measures, only pleural line abnormalities, finding remained at high risk of publication bias. We used the QUADAS2 tool to assess the study quality and risk of bias; the included studies mostly revealed an unclear or moderate risk of bias (Supplemental Material Table 1). There was marked heterogeneity with regard to pooling the proportion of pleural thickening, consolidation, or pleural effusion.

## DISCUSSION

COVID-19 has struck the world with surprise, resulting in elated morbidity and mortality, strained the healthcare system, and depleted the resources even in resource-rich settings.^19^ Up to the date of submitting this manuscript, COVID-19 has affected 3 million individuals (confirmed cases) and resulted in more than 200,000 deaths worldwide.^20^ Tools to aid in the early identification and follow-up are needed in an attempt to provide appropriate care and to allocate resources better.^21,22^

Computed tomography scan has surfaced as a useful imaging modality in the diagnosis and follow-up of COVID-19.^23,24^ Although useful, its use is limited, as explained earlier. The recent interest in point-of-care ultrasound (POCUS) of the lungs is due to its portability, steep learning curve, a relatively easier sterilization process, absence of ionizing radiation exposure, and its role in the follow-up. Moreover, it has an excellent correlation with CT scan in various pulmonary diseases (B-lines, subpleural consolidations, and irregular pleural line).^4,25^ The use of POCUS is of greater value in resource-limited settings, for example, in some tropical areas where other diagnostic modalities may not be readily available, and testing resources may be limited. In these settings, basic ultrasound image acquisition and interpretation skills can be taught to healthcare providers of varying experiences, following a brief training course.^26^

In our review, B-pattern predominated, occurring with a pooled frequency (PF) of 97% (94–100%). The results were consistent and homogenous across all the constituent studies. Pleural line abnormalities were present in two-thirds of the cases (PF 70%, 95% CI: 13–100%), the frequency of other findings was less in our review, and the results were extremely heterogeneous. This heterogeneity is likely owing to differences in the settings, patient populations, number of lung zones examined, the level of operator expertise, ultrasound machine or probe used, stage and severity of the illness. Hence, the presence of these findings (consolidations, pleural thickening, or pleural effusion) may be useful in the triage, prognosis, and follow-up. However, their absence cannot be used to rule out COVID-19. Two of the constituent studies (Guorong et al.^18^ and Poggiali et al.^9^) demonstrated a good correlation between the LUS and CT scan findings. Furthermore, Guorong et al. demonstrated that the LUS findings improve synchronously with clinical improvement, suggesting a potential role of LUS in the clinical follow-up. However, this role needs to be supported by future studies.^9,18^

It is worthy to note two studies that were excluded from our review. The first study examined the role of lung US in asymptomatic patients with COVID-19, hence excluded.^27^ In their retrospective analysis of nine asymptomatic patients, LUS revealed abnormalities in 22% (*n* = 2/9). One patient had B-pattern, and the other patient had pulmonary consolidations. Whereas the frequency of B-pattern was low in this study, CT scan did similarly depict abnormalities in only 33% (*n* = 3/9), indicating a possible low yield of various imaging modalities in asymptomatic COVID-19 patients. The second study was excluded as it was limited to a pediatric cohort. They retrospectively analyzed the data of eight COVID-19–infected children; 88% (*n* = 7/8) had abnormalities on lung US (B-pattern *n* = 5/8, consolidations *n* = 2/8). On clinical improvement, all the lesions radiologically improved either partially or entirely, hinting toward a potential role of US in the clinical follow-up.^28^

In the 2009 H1N1 pandemic, LUS findings were used to differentiate between viral and bacterial pneumonia with an excellent interobserver agreement. Bacterial pneumonia findings were lung consolidations with sonographic air bronchograms. However, the findings noted in cases of viral pneumonia were similar to our findings (B-pattern, pleural line abnormalities, or subpleural consolidations).^8,26^ It may be argued that LUS findings may not enable clinicians to differentiate COVID-19 from other viral lung infections; however, having such a prevalent finding amid a pandemic will lead to faster diagnostic and therapeutic decisions and better resource allocation.

Our review aimed to pool the reported proportions of various findings detected by POCUS lungs and is the first meta-analysis aimed at assessing the role of lung US or POCUS in the diagnosis of COVID-19. The authors are of the view that this will be of value to frontline clinicians. We believe that the findings from our review will assist in the integration of this useful modality in the triage, diagnosis, management, and follow-up of COVID-19 patients.

Our review is limited by a small number of constituent studies, a small number of patients, unclear bias risk, and inability to rule out publication bias. Also, there is a lack of unifying definitions and inconsistencies in the reporting of various lung abnormalities. Inadequate reporting of the extensiveness of LUS findings (lung areas involved or a representative LUS score) may limit its role in the temporal follow-up and its prognostic value. Finally, we were not able to calculate the sensitivity and specificity owing to the absence of data necessary for their computation.

A well-conducted diagnostic accuracy study comparing LUS, CT scan, and various specific tests for COVID-19 (PCR, IgM, and IgG on serial measurements) to ascertain the sensitivity, specificity, and diagnostic accuracy of each modality in the diagnosis of COVID-19 is needed. In addition, we suggest studying LUS on various severity spectrum of the disease to identify findings that correlate with disease severity. The results of the recently planned and ongoing trials, such as POCUSCO, ECHOVID-19, POCUSars-CoV-2, VIRUS, and COVILUS, will address some of the aforementioned limitations.^29–33^

## CONCLUSION

Evidence of interstitial lung involvement, as depicted by B-pattern, is the most common and consistent finding on lung US in COVID-19 patients. Although nonspecific, the presence of this finding amid the COVID-19 pandemic, in addition to other characteristic symptoms, will increase the disease likelihood. Thus, POCUS will likely play a vital role in the future triage, diagnosis, management, and follow-up of COVID-19 patients.

## Supplemental table and figure

Supplemental materials
