# Supplementary material for: Frequency of Abnormalities Detected by Point-of-Care Lung Ultrasound in Symptomatic COVID-19 Patients: Systematic Review and Meta-Analysis
Source: Am J Trop Med Hyg. 2020 Jun 2;103(2):815–21. doi: 10.4269/ajtmh.20-0371 (PMC7410428; doi:10.4269/ajtmh.20-0371)
Supplement: Supplementary file 1 [file tpmd200371.SD1.pdf]

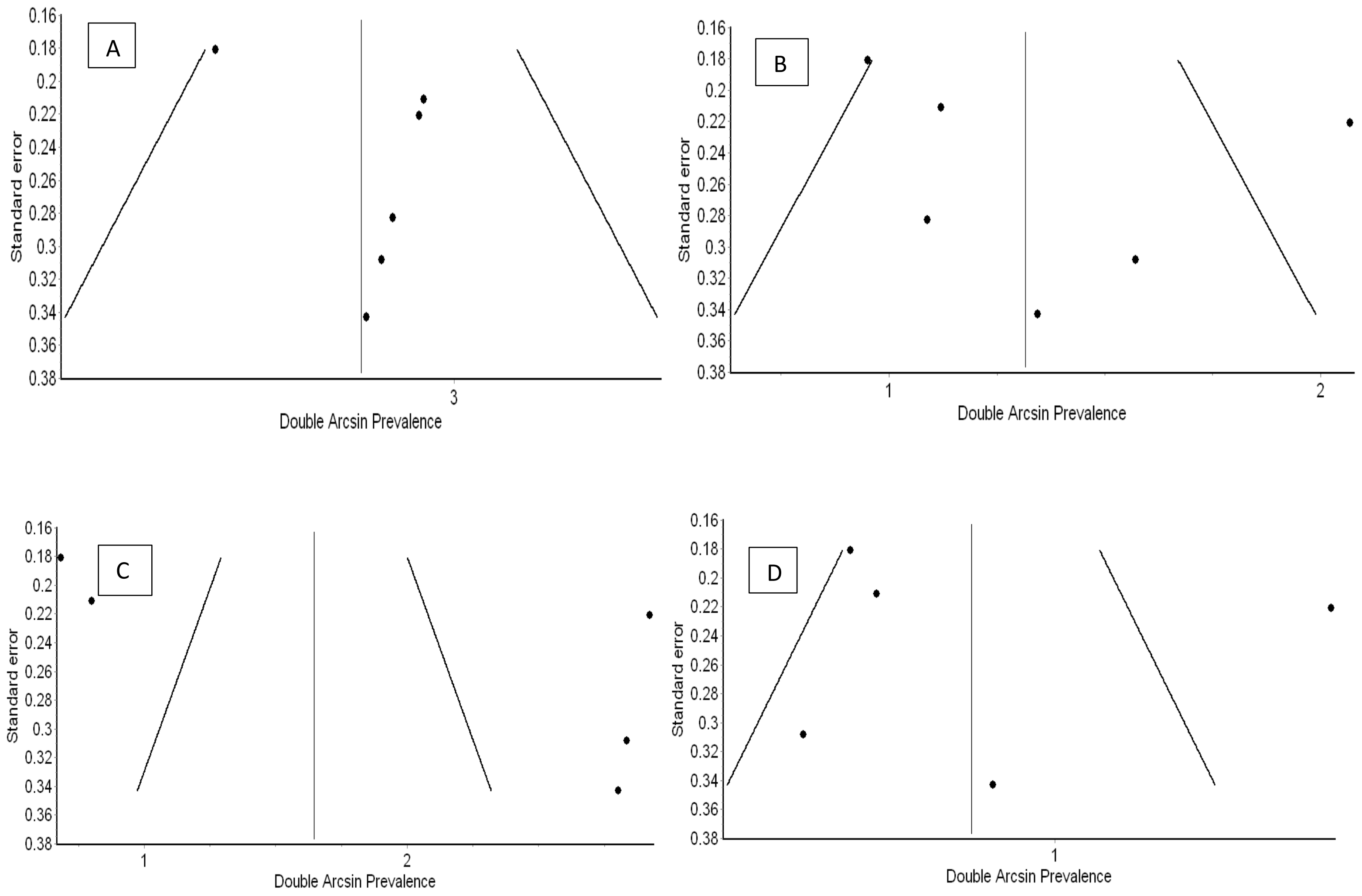

Supplementary Figure 1: Funnel plots to ascertain the publication bias in (A) B-pattern, (B) consolidations, (C) pleural line abnormalities, and (E) pleural effusion. It revealed potential publication bias in (C). Comparative DOI plots with LFK indices were populated for comparison. It showed no publication bias for (A) while showing a minor risk for (B), (E).

Study	Risk of bias				Applicability concerns		
	Patient Selection	Index Test	Reference Standard	Flow and Timing	Patients Selection	Index Test	Reference Standard
Lomoro 2020	?	?	😃	?	?	?	😃
Peng 2020	?	?	?	?	?	?	?
Huang 2020	😃	😃	😃	?	😃	😃	😃
Poggiali 2020	?	?	?	?	?	?	😃
Lu 2020	?	😃	😃	😃	?	😃	😃
Lyu 2020	?	😃	😃	?	?	😃	😃
Yasukawa 2020	?	😃	😃	😃	😃	?	😃

Table S1: Quality assessment of study included in the review (QUADAS2)

The reference test here is the diagnostic tests confirming the diagnosis of COVID-19. Most patients did not undergo CT scan simultaneously or at all. Most studies had unclear bias risk.

😃 Low risk of bias

☹ High risk of bias

? unclear risk of bias.
